# Device-based physical activity measures for population surveillance—issues of selection bias and reactivity

**DOI:** 10.3389/fspor.2023.1236870

**Published:** 2023-08-08

**Authors:** Lars Breum Christiansen, Sofie Koch, Adrian Bauman, Mette Toftager, Christina Bjørk Petersen, Jasper Schipperijn

**Affiliations:** ^1^ Department of Sports Science and Clinical Biomechanics, Faculty of Health Sciences, University of Southern Denmark, Odense, Denmark; ^2^ Charles Perkins Centre, The University of Sydney, Camperdown, NSW, Australia; ^3^ School of Public Health, Faculty of Medicine and Health, The University of Sydney, Sydney, NSW, Australia; ^4^ National Institute of Public Health, Faculty of Health Sciences, University of Southern Denmark, Copenhagen, Denmark

**Keywords:** representativity, recruitment, monitoring, accelerometry, hawthorne effect

## Abstract

**Background:**

Device-based measurement in physical activity surveillance is increasing, but research design choices could increase the risk of self-selection bias and reactive behaviour. The aim of this study is to compare the self-reported physical activity profiles of four different samples: participants in a large national survey, participants in a telephone-based survey of non-responders, participants in the large national survey who accepted the invitation to device-based measuring, and the same sample during the week of monitoring.

**Methods:**

In October 2020, 163,133 Danish adults participated in a national survey and of those 39,480 signed up for device-based measurements. A balanced random sample (*n* = 3,750) was invited to wear an accelerometer of whom 1,525 accepted the invitation. Additionally, a short telephone-based survey on 829 non-responders to the national survey was conducted. Sociodemographic characteristics and self-reported weekly frequencies of physical activity across four domains are compared.

**Results:**

The participants in the national survey were older, more often female, and more often not working. Participants in the telephone-based survey were younger, more often doing unskilled work, and were more often active at home and at work. The participants in the device-based sample were more often active during transport and leisure in the national survey, and participants categorized in the most active category increased during the week of monitoring from 29.0% to 60.7% and from 58.5% to 81.7% for active transport and leisure activities, respectively.

**Conclusion:**

Recruiting a population representative sample for device-based measurement of physical activity is challenging, and there is a substantial risk of sample selection bias and measurement reactivity. Further research in this area is needed if device-based measures should be considered for population physical activity surveillance.

## Introduction

Physical activity surveillance is needed to assess population trends over time and progress towards achieving the WHO 2030 physical activity goals (1). The principles of surveillance include comprehensive, regular assessment of physical activity and related indicators using representative population samples. Although device-based measures reduce measurement error in assessing physical activity (2), they do not collect contextual information, and methods for data processing are not yet standardised (3–6). One issue that has received little attention is the potential for self-selection bias in the samples that provide accelerometer data, and this may reduce representativeness, which is a major concern in generalising to population estimates (7, 8). Another issue is measurement reactivity, which induces another form of bias (9–11). In a large population study in Denmark, we observed findings related to these possible biases. The aim of this study is to compare the self-reported physical activity profiles of four different samples: participants in a large national survey, participants in a telephone-based survey of non-responders, participants in the large national survey who accepted the invitation to device-based measuring, and the same sample during the week of monitoring. This insight into bias due to study design is an important contribution to the debate around using device-based measures in population-based surveillance.

## Methods

**Figure 1:**
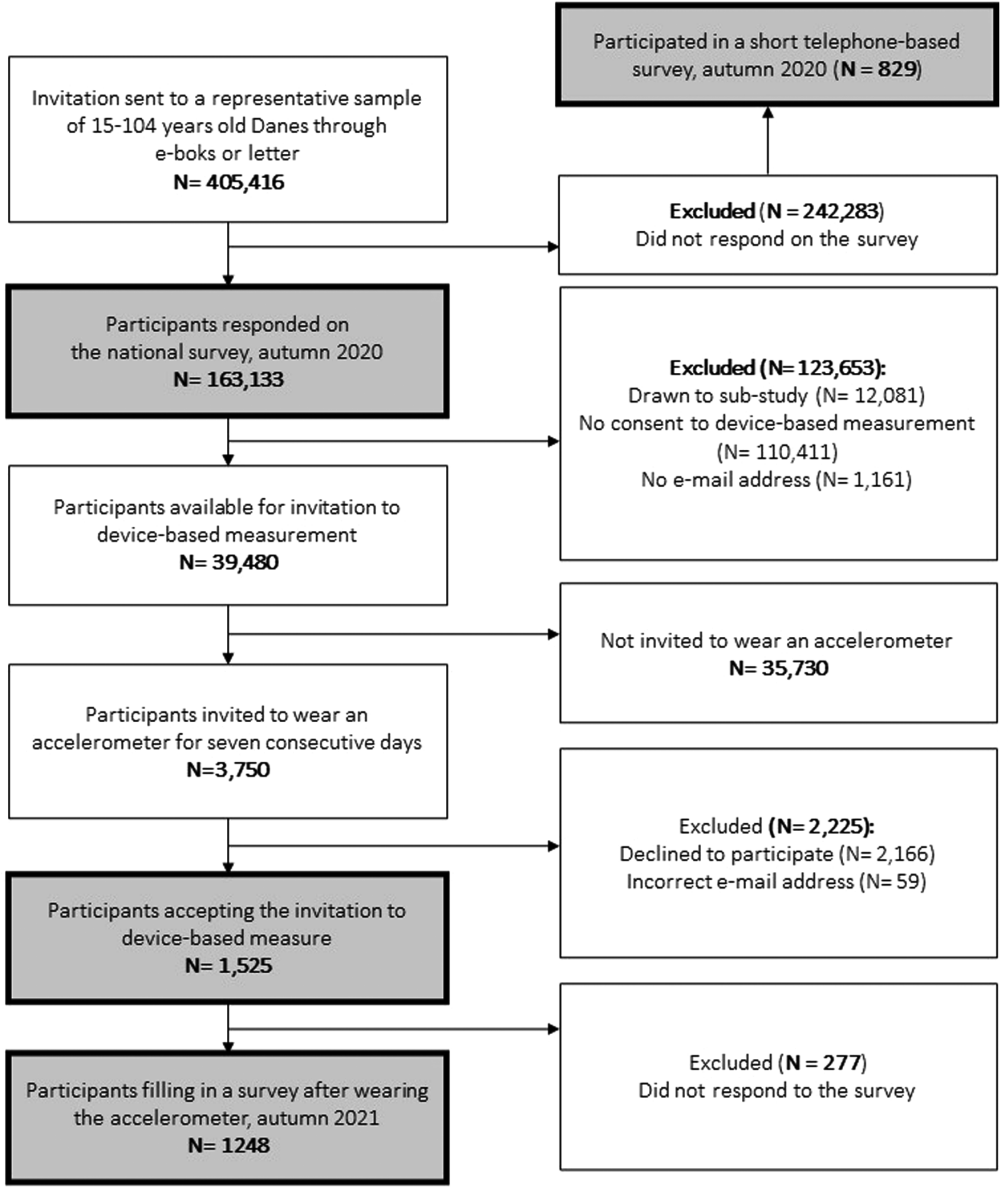
Flowchart of recruitment in *the moving Denmark study.*

*The Moving Denmark Study* is a large national survey of physical activity patterns and behaviours combined with device-based measurements in a subsample (Figure 1). A representative sample of 405,416 Danish adults (15 + years) were invited to participate in October 2020, with 163,133 people responding to the questionnaire (response rate 40%). At the end of the survey respondents were offered the opportunity to participate in sub-studies, including a study of device-based measured physical activity. Of the 163,133 respondents, 39,480 signed up for the device-based measurements. In June 2021, a balanced random sample based on the distribution of sex and age of the original representative sample was invited via e-mail to wear an accelerometer for seven consecutive days. A total of 3,750 invitations were sent of whom 1,525 accepted the invitation, and 1,248 completed a questionnaire after wearing the accelerometer. Further, we conducted a short telephone-based survey on a sample of 829 non-responders to the national survey. The study and its data-management procedures were approved by the Research & Innovation Organization of the University of Southern Denmark (No. 10.680). All respondents were informed about the study and that their participation was voluntary, and they could withdraw at any time.

**Figure 2:**
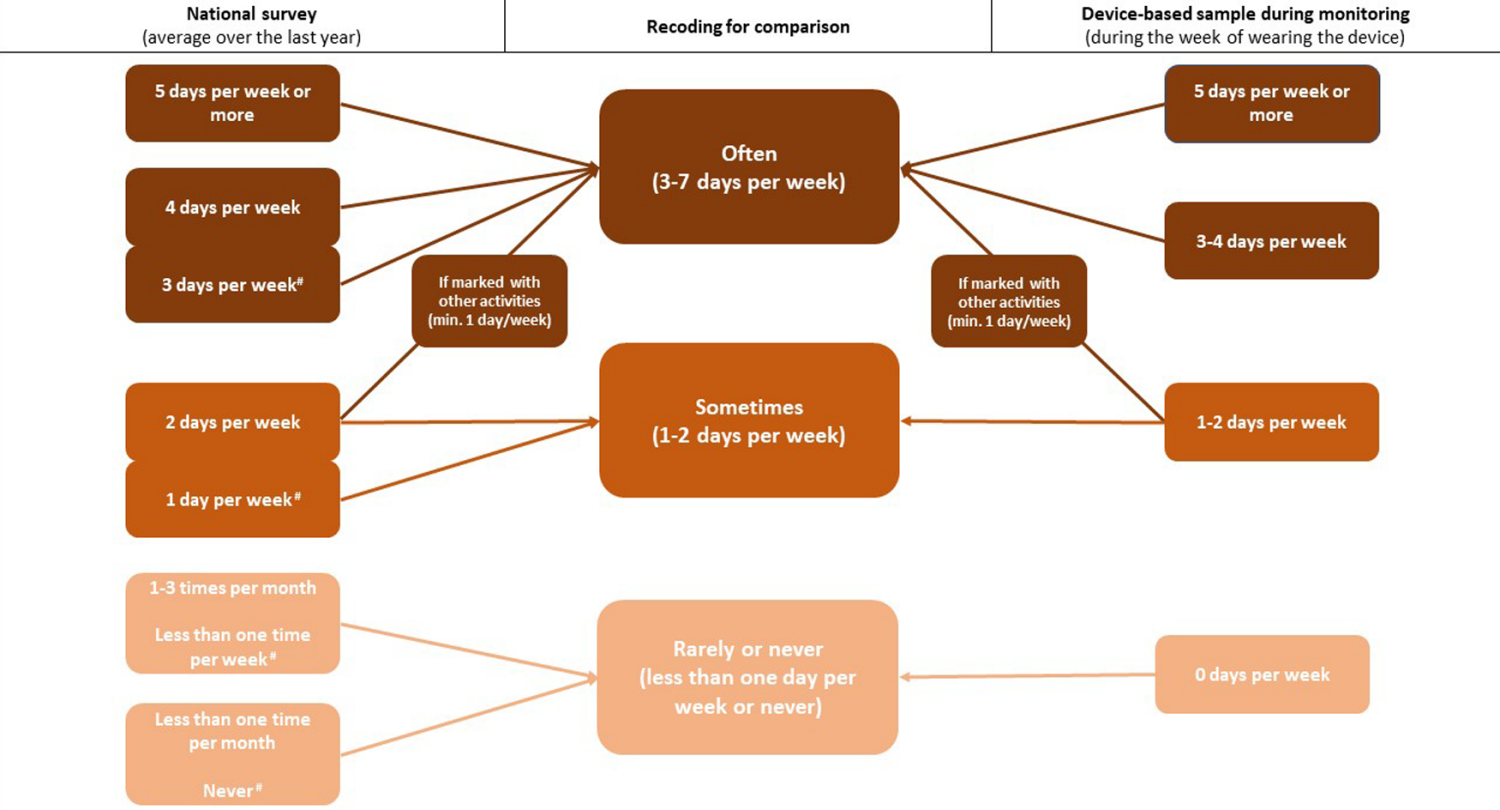
Visualization of the recoding of physical activity frequencies from the national survey over the last year and the survey concerning the seven days of device-based monitoring. #: For the domains home, work, and active transport to work and leisure the response categories “3 and 4 days”, “1 and 2 days” were joined in one. For those domains the response categories for least often were less than one time per week and never.

All three samples answered newly developed questions regarding self-reported weekly frequencies of physical activity across five domains: at home, at work or education, during transportation (to work/education and to other destinations), and during leisure time. In each of the five domains, the respondents were asked to consider the last year, and indicate how often they participated in specific activities with moderate to high intensity within each domain on a weekly basis (Supplementary Table S1). For each domain the responses were aggregated into three categories. The *often* category were those reporting one or more physical activities with moderate to high intensity on three or more days per week. The two other categories were labelled “sometimes” and “rarely” and were those reporting one or more physical activities with moderate to high intensity 1–2 days per week and less than one time per week, respectively (Figure 2, left side).

The participants for device-based measurement were mailed an Axivity AX3 accelerometer and were asked to wear it taped to their thigh for seven consecutive days. The accelerometer data were collected between August and October 2021. Immediately after wearing the accelerometer, participants were asked to complete a final survey to assess self-reported physical activity frequencies across the five domains during the week of device-based measurement. This survey resembled the one they filled in for the national survey except for the recall period being only the week of device-based measurement. This contrasted with the national survey, where respondents recalled the weekly average over the last year (Figure 2, right side). Despite the differences, it is possible to compare the weekly frequencies of self-reported physical activity among three samples (national survey, non-responders and device-based sample), and for the participants in the device-based subsample at two time points (national survey and after device-based measurement). The confidence intervals of the distributions were calculated, and non-overlapping intervals were inferred as a significant difference.

## Results

The distribution of sample sociodemographic characteristics in the three samples are presented in Table 1. The participants in the telephone-based survey of non-responders were younger (45.4 years vs. 49.7–51.3 years) and more often working in unskilled or vocational jobs (30.2% vs. 20.0%–21.7%). The participants in the national survey were older (51.3 years vs. 45.4–49.7 years), more often female (54.6% vs. 45.5%–52.5%), and they were more often not working (retired or unemployed) compared to the other samples (34.0% vs. 24.5%–28.8%). The participants in the device-based sample were most often working in jobs requiring higher education or self-employed (36.7% vs. 29.7–33.2). Due to an overrepresentation of older and female participants accepting the invitation to participate in the device-based measures, we used a block randomisation of groups based on age and sex. This explains the small age and sex difference between the two samples.

**Table 1:** Sociodemographic characteristics of the groups.

Characteristics	Telephone-based survey to non-responders to the national survey (*N *= 829)	National survey participants (*N = *163,133)	Device-based sample at national survey (*N = *1,525)
Age, years (SD)	45.4 (SD 17.8)	51.3 (SD 18.4)	49.7 (SD 17.8)
Sex, female (%)	45.5	54.6	52.5
Occupational status			
Working jobs requiring higher education or self-employed (%)	33.2	29.7	36.7
Working in unskilled or vocational jobs (%)	30.2	21.7	20.0
Not working (%)	24.5	34.0	28.8
In education (%)	8.8	10.1	9.9
Other (%)	3.4	4.6	4.6

**Figure 3:**
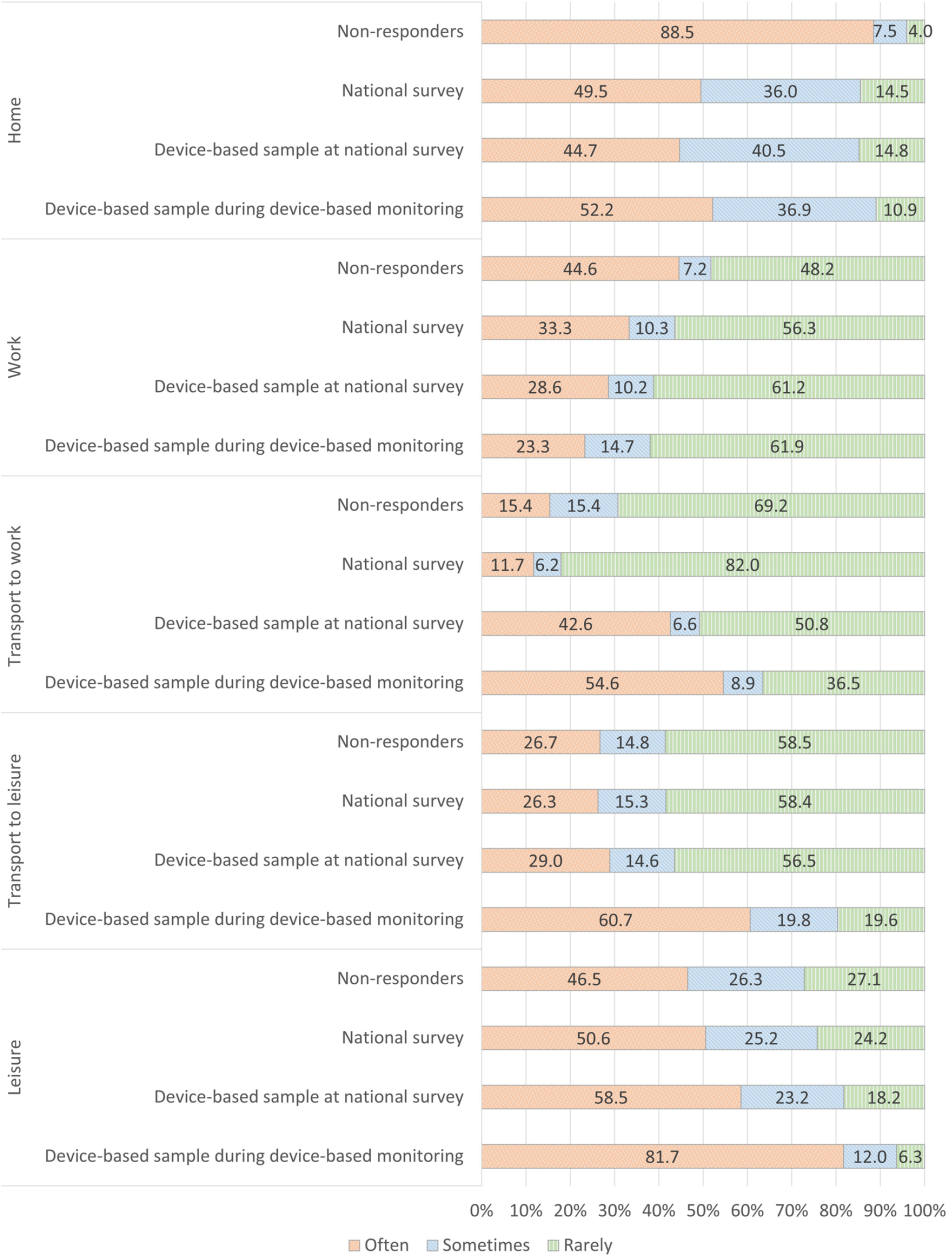
Aggregated frequencies of weekly physical activities across domains and samples.

In Figure 3, we present the proportion of respondents in three frequency categories within the five domains of physical activity (see Supplementary Tables S1, S2 for additional information).

For the domestic physical activity domain, we observe a large significant difference between the non-responders participating in the short telephone-based survey (Often: 88.5%) and the other three samples (Often: 44.7%–49.5%). For physical activity at work, we observe a similar difference, where more non-responders are categorized in the most active category (44.6% vs. 23.3%–33.3%). The participants of the device-based monitoring are less active in this domain (Often: 28.6%), which is even lower during the week of monitoring (Often: 23.3%).

For the active transport to work domain, we observe a large significant difference between the sample who accepted the invitation to complete device-based measurement (Often: 42.6%) and the non-responders (Often: 15.4%) and the large sample in the national survey (Often: 11.7%). This difference increases during the week of monitoring (Often: 54.6%). For active transport to leisure activities, there are no significant differences between the non-responders and the two samples in the large national survey (Often: 26.3%–29.0%). However, 60.7% of the participants are categorized in the most active category during the week of monitoring. In the leisure activity domain, a gradual difference is observed. Fewest of the non-responders are categorized as often active (46.5%), which increases to 50.6% for the national survey, 58.5% for those accepting the invitation to device-based measures, and 81.7% during the week of monitoring.

## Discussion

In the current study, the device-based sample was recruited by an invitation in the end of the questionnaire of a large survey related to physical activity behaviour, and even though the accelerometers were blinded, we offered feedback to the participants of their results after the week of monitoring. Comparing the self-reported physical activities in the four samples, we observed large differences among the samples, which led us to speculate that the device-based sample may be a more active and motivated group, and thus be more prone to reactive behaviour. The findings raise general questions regarding selection and reporting effects when using different modes of measurements for population-based surveillance of physical activity. The variations in self-reported physical activity are due to the measures used, but also the mode of recruitment and final samples obtained.

The participants who indicated interest in participating in device-based activity measurement were older and more often female, and therefore we balanced our invited subsample the known age and gender distribution in the background population. We considered balancing the invitations using the survey answers on physical activity but refrained from doing so due to a concern that it might further influence representativeness. Recruiting our accelerometer sample from those 40,000 survey respondents completing the survey and stating interest in wearing a device is likely to have increased participation numbers in the device-based subsample, but also may have increased selection bias towards a more active population. Approximately one third of the respondents were initially interested, 40% confirmed their interest when they received the invitation 9 months later with more details of the device-based measurements. An issue here may be the perceived respondent burden in accelerometry assessment. An alternative solution would have been to recruit an independent sample for the device-based monitoring study alone.

Another issue to consider is the concealment of data. We used blinded accelerometers (i.e., respondents could not see how active they were), but in order to encourage recruitment, we offered the respondents a summary of their data afterwards. In essence, they were aware that they were being observed, and that they would receive delayed feedback of their physical activity behaviour. In a study of reactivity to pedometers, Clemens and Parker (9) compared sealed, unsealed and diary with a covert condition (blind to the aim). The participants were least active during the covert condition and increased their activity in each of the three other conditions from sealed to diary. The effect of feedback is supported by the review of König et al. (11), who found that measurement reactivity is more pronounced if the measurement is observable by respondents. In the review, several studies found no evidence of reactivity, but typically those studies have used other sampling and recruitment methods and do not take motivation for physical activity into consideration. The issue of reactivity might be exacerbated if the respondent has social desirability towards the measured behaviour e.g., to be physical active (10, 11). Although this is a possible bias, we have no direct evidence that our feedback influenced measurement reactivity. Future studies should investigate how different feedback incentives in device-based measurements affect recruitment and reactivity.

To minimize the risk of reactive behaviour, participants should ideally be blinded to the measurement without knowing the exact purpose of the device, which causes ethical and practical dilemmas. In the pedometer study by Clemens and Parker (9) the participants were at first informed that the pedometer was a “body posture monitor”, which resulted in less physical activity compared with the other conditions. Masking the real aim of the study is also highlighted as a possibility in a recent guide to minimizing measurement reactivity in trials (10). Combined monitors measuring behaviours or physical indicators (sleep, heart rate, elevation etc.) might also diminish reactivity concerning a specific (socially desirable or undesirable) behaviour.

To increase population representativeness, the device-based measurement needs to be feasible for the entire population (low cost and low participant burden). Technological development of inobtrusive, smarter and cheaper devices might help solve this problem. At the same time, the duration of measurement should also be considered. Measurement reactivity is more likely if the behaviour is easy to change, e.g., light intensity physical activity (12), is socially desirable for the participant and should be sustained for a short period of time (11). Research shows attenuation of measurement reactivity after the first days (11, 12), but studies have found increased physical activity in the end of the week of pedometer monitoring (9). In order to minimise reactivity, it has been advised to prolong the monitoring period and exclude the first week (9, 11). However, a longer measurement period could increase the participant burden and possibly selection bias.

We recognise that this study has several limitations including comparability issues between the samples. First, the national survey was conducted in the Autumn of 2020 and device-based measurements were collected in Autumn 2021. At the end of 2020, the COVID-19 situation in Denmark was stable and sport facilities were open. Large scale vaccination programmes had not yet been initiated, there were restrictions on large gatherings, and facemasks were mandatory in all public indoor spaces. By the end of 2021, all shops, services, and sport facilities had reopened, but face masks were still required indoors, and the majority of the population had received COVID-19 vaccinations. The fact that the data were collected at different time points might explain some of the differences in reported physical activity during the device-based measurement period.

Secondly, there are differences in the recall period for the questions regarding physical activities. In the national survey and in the telephone-based survey, the respondents were asked about the weekly frequency of activities during the last 12 months, while the participants of the device-based measurements were asked to report their behaviour for the week that they wore the accelerometer. This difference could affect the comparability between the estimates, even though we asked respondents to disregard seasonal off-periods where they were not doing the specific activity.

Thirdly, we should highlight that this comparison is based on the analysis of self-reported data, which previously have been found to be incomparable to direct measurement (13). Differences between samples could be due to both real differences in behaviour and differences in questionnaire completion. Responses to questions for physical activities in the previous week where the participants know they are being measured may be reported differently to responses regarding average weekly participation in activities during the previous 12 months. One could argue that the measurement makes participants more aware of a behaviour, which improves their ability to remember and thus will increase the frequency of activities they report. That is not the case for work-related physical activities. Additionally, there might also be a difference in reporting between telephone-based survey for the non-responders and online survey for the respondents.

Collecting accelerometer data in large samples is feasible, but as our results posit, recruiting a population-representative sample to answer a questionnaire and then to wear a device is a challenge. We do not know if our results would have been different if we had drawn a second, independent random sample instead of inviting them as a sub-sample from the large survey, but it is possible that the response rate in a random population sample would be lower and maintain or exacerbate selection effects. It is important to consider the challenges in recruitment of representative samples for population surveillance vs. the benefits of device-based assessments. This debate has implications for physical activity surveillance systems, and for debates regarding the trade-off between more accurate (device-based) measurement, but at the cost of less generalisable population estimates.

## Conclusion

Based on the experiences from the Moving Denmark study we address selection biases which affect the generalisability of device-based physical activity estimates in population-based surveillance. In comparing different samples, we found differences regarding sociodemographic characteristics and especially physical activity behaviours, which made us reflect on methodological challenges in collecting device-based measures for physical activity surveillance. The sample who accepted the invitation for device-based measurements were more active in the active transport and leisure domains, and this difference was increased during the week of device-based measurement. We acknowledge that this study has several methodological limitations, and that the observed differences could be amplified by our mode of recruitment or by measurement itself, and results should therefore be interpreted with caution. We hope these findings can stimulate and qualify the debate on reporting device-based population estimates and initiate further research into improving the generalisability of prevalence estimates in physical activity surveillance.

## Data Availability

The raw data supporting the conclusions of this article will be made available by the authors, without undue reservation.
