# Supplementary material for: Device-based physical activity measures for population surveillance—issues of selection bias and reactivity
Source: Front Sports Act Living. 2023 Aug 8;5:1236870. doi: 10.3389/fspor.2023.1236870 (PMC10442809; doi:10.3389/fspor.2023.1236870)
Supplement: Supplementary file 1 [file Table1.docx]

**Supplementary Table 1:** Physical activities of medium and high intensity in the five domains included in the aggregated indexes of physical activity (Figure 2).

| **Domain (question)** | **Physical activity of medium to high intensity** |
| --- | --- |
| **Home:**  How often do you perform the following types of practical work at home? | Cleaning |
|  | Gardening |
| **Work and study:**  How often is your work or studies characterized by? | Tasks that require moderate physical effort |
|  | Tasks that require heavy physical effort |
| **Transport to work or education:**  How often do you use the following modes of transport on the way to and from your work or place of study? | Cycling (incl. electric bike) |
|  | Walk |
|  | Run |
| **Transport to other destinations:**  How often do you use the following modes of transport to and from shopping, institutions, leisure activities, visiting friends and family and the like? | Cycling (incl. electric bike) |
|  | Walk |
|  | Run |
| **Leisure:**  How often have you performed the following activities within the last 12 months? If there are large fluctuations in how often you performed the activities during the year, please indicate an average for the period in which you performed the activities? | Gymnastics |
|  | Activities on water |
|  | Outdoor activities |
|  | Physically active games and games |
|  | Walking and hiking tours (not as transport) |
|  | Other ball games |
|  | Cycling (excluding transport) |
|  | Fitness and physical training |
|  | Dance |
|  | Roller and street activities/street sports |
|  | Other sporting activities |
|  | Run (excluding transport) |
|  | Team ball games |
|  | Activities in water |
