# Supplementary material for: Device-based physical activity measures for population surveillance—issues of selection bias and reactivity
Source: Front Sports Act Living. 2023 Aug 8;5:1236870. doi: 10.3389/fspor.2023.1236870 (PMC10442809; doi:10.3389/fspor.2023.1236870)
Supplement: Supplementary file 2 [file Table2.docx]

**Supplementary Table 2:** Aggregated frequencies of weekly physical activities across domains and groups with confidence intervals (Figure 3)

| **Domain** | **Sample** | **Often** | **Sometimes** | **Rarely** |
| --- | --- | --- | --- | --- |
| **Leisure** | Device-based sample during device-based monitoring (n=1,248) | 81,7  (79,4-83,8) | 12,0  (10,2-13,9) | 6,3  (5,0-7,8) |
|  | Device-based sample at national survey (n=1,530) | 58,5  (56,0-61,1) | 23,2  (21,1-25,4) | 18,2  (16,3-20,2) |
|  | National survey (n=150,673) | 50,6  (50,3-50,9) | 25,2  (25,0-25,4) | 24,2  (24,0-24,4) |
|  | Non-responders (n=829) | 46,5  (43,1-50,0) | 26,3  (23,3-29,4) | 27,1  (24,1-30,3) |
| **Transport to leisure** | Device-based sample during device-based monitoring (n=1,248) | 60,7  (57,9-63,4) | 19,8  (17,6-22,1) | 19,6  (17,4-21,9) |
|  | Device-based sample at national survey (n=1,530) | 29,0  (26,7-31,3) | 14,6  (12,9-16,5) | 56,5  (54,0-59,0) |
|  | National survey (n=161,274) | 26,3  (26,1-26,5) | 15,3  (15,1-15,5) | 58,4  (58,2-58,6) |
|  | Non-responders (n=829) | 26,7  (23,7-29,9) | 14,8  (12,5-17,4) | 58,5  (55,1-61,9) |
| **Transport to work** | Device-based sample during device-based monitoring (n=911) | 54,6  (51,3-57,9) | 8,9  (7,1-10,9) | 36,5  (33,4-39,7) |
|  | Device-based sample at national survey (n=1,019) | 42,6  (39,5-45,7) | 6,6  (5,2-8,3) | 50,8  (47,7-53,9) |
|  | National survey (n=98,730) | 11,7  (11,5-11,9) | 6,2  (6,0-6,4) | 82,0  (81,8-82,2) |
|  | Non-responders (n=597) | 15,4  (12,6-18,5) | 15,4  (12,6-18,5) | 69,2  (65,3-72,9) |
| **Work** | Device-based sample during device-based monitoring (n=911) | 23,3  (20,6-26,2) | 14,7  (12,5-17,2) | 61,9  (58,7-65,1) |
|  | Device-based sample at national survey (n=1,019) | 28,6  (25,8-31,5) | 10,2  (8,4-12,2) | 61,2  (58,1-64,2) |
|  | National survey (n=99,081) | 33,3  (33,0-33,6) | 10,3  (10,1-10,5) | 56,3  (56,0-56,6) |
|  | Non-responders (n=597) | 44,6  (40,6-48,7) | 7,2  (5,3-9,6) | 48,2  (44,1-52,3) |
| **Home** | Device-based sample during device-based monitoring (n=1,248) | 52,2  (49,4-55,0) | 36,9  (34,2-39,6) | 10,9  (9,2-12,8) |
|  | Device-based sample at national survey (n=1,530) | 44,7  (42,2-47,2) | 40,5  (38,0-43,0) | 14,8  (13,1-16,7) |
|  | National survey (n=163,087) | 49,5  (49,3-49,7) | 36,0  (35,8-36,2) | 14,5  (14,3-14,7) |
|  | Non-responders (n=829) | 88,5  (86,1-90,6) | 7,5  (5,8-9,5) | 4,0  (2,6-5,6) |
